# Supplementary material for: Structure induced laminar vortices control anomalous dispersion in porous media
Source: Nat Commun. 2022 Jul 2;13:3820. doi: 10.1038/s41467-022-31552-5 (PMC9250523; doi:10.1038/s41467-022-31552-5)
Supplement: Supplementary file 1 — Supplementary Information [file 41467_2022_31552_MOESM1_ESM.pdf]

SUPPLEMENTARY INFORMATION FOR
Structure induced laminar vortices control anomalous dispersion in porous media

Ankur Deep Bordoloi,[1] David Scheidweiler,[1] Marco Dentz,[2]
Mohammed Bouabdellaoui,[3] Marco Abbarchi,[3] and Pietro de Anna[1]

[1] *Institute of Earth Sciences, University of Lausanne, Lausanne 1015, Switzerland*
[2] *Spanish National Research Council (IDAEA-CSIC), Barcelona 08034, Spain*
[3] *Aix Marseille Univ, Université de Toulon, CNRS, IM2NP 13397, Marseille, France*

I. SUPPLEMENTARY INFORMATION I

Molds were fabricated by depositing a layer of SU-8 2150 (MicroChem Corp., Newton, MA) with controlled thickness of 0.083 mm on silicon wafers via spin-coating and the desired geometry was engraved via photolithography. Polydimethylsiloxane (PDMS; Sylgard 184 Silicone Elastomer Kit, Dow Corning, Midland, MI) was prepared with an addition of 10% by weight of curing agent and casted on the molds. PDMS microchannels were plasma-sealed onto 25×75 mm glass slides [1].

II. SUPPLEMENTARY INFORMATION II

We independently measure the diffusion coeffcient (D) for 0.5 μm DAPI colloids in the same confined geometry used in the experiment tracking suspended particles under no flow condition. We capture a time sequence of fluorescence images of suspended particles at $1/10^{\text{th}}$ of a second for 100 seconds. A small subsection of visualized particles is shown in Supplementary Fig. 3a. The sample trajectories shown in Supplementary Fig. 3b suggest that the particles have nearly negligible mean displacement. The diffusion coefficient is computed based on 7296 independent trajectories from the slope of the mean-square displacement MSD $= \left\langle (r - \langle r \rangle)^2 \right\rangle$, which is expected to scale as $2dDt$, where $r = \sqrt{(X - X(0)^2 + (Y - Y(0)^2}$ and $d = 3$ is the dimensionality of the system (see Supplementary Fig. 3c).

The diffusion coefficient of a colloidal particle in the bulk of a fluid can be estimated using the Stokes-Einstein equation:

$$D = k_B T / 6\pi\mu r, \tag{1}$$

where $k_B = 1.381 \times 10^{-23} \text{m}^2\text{kgs}^{-2}\text{K}^{-1}$ is the Boltzmann constant, $T = 298K$ is the absolute colloidal temperature, and $\mu = 1.1 \times 10^{-3}$ Pa.s is the viscosity of the liquid (a milliQ water-D_2O mixture 1:1). For the particles (radius, $r = 2.5 \times 10^{-7}$) used in the experiment the theoretical prediction is $D = 7.94 \times 10^{-7} \text{mm}^2/\text{s}$. The experimentally measured diffusion coefficient $D = 1.4 \times 10^{-7} \text{mm}^2/s$ for the confined medium is 5.6 times smaller than the value predicted in equation 1 as expected, since the colloids cannot be considered suspended in the bulk due to the close proximity to no-slip boundary at the grain walls.

III. SUPPLEMENTARY INFORMATION III

We consider here the trapping times in a vortex that is confined at the vertical boundaries by solid walls and open at the horizontal boundaries, see Supplementary Fig. 8. In our discussion, we follow the analyses of [2] and [3]. To this end, we consider the advection-diffusion (mass conservation) equation for the solute concentration $c(\mathbf{x}, t)$,

$$\frac{\partial c(\mathbf{x}, t)}{\partial t} + \mathbf{v}(\mathbf{x}) \cdot \nabla c(\mathbf{x}, t) - D\nabla^2 c(\mathbf{x}, t) = 0, \tag{2}$$

for an instantaneous solute pulse $c(\mathbf{x}, t = 0) = \delta(t)$ at the upper boundary of the roll. The solute advects and diffuses into the convection roll. The trapping time is given by the solute return time distribution from the vortex to the upper boundary. For pure diffusion, i.e., for $\mathbf{v}(\mathbf{x}) \equiv \mathbf{0}$, the trapping time distribution scales as $\phi(t) \sim t^{-3/2}$ [4]. The same behavior would be obtained if the flow in the vortex were simply a solid body rotation, see also below. Here this is different due to the shear flow along the solid boundaries. The distance between streamlines is larger in the shear flow close to the solid boundaries than at the horizontal boundaries, see Supplementary Fig. 8. This means, the distance a particle diffuses inside the vortex across streamlines at the vertical boundary, is decreased at the horizontal boundary due to the compression of the streamlines. This interplay leads to a steeper decay of the trapping time distribution as $\phi(t) \sim t^{-5/3}$ because particles have the chance to leave the vortex earlier than in the absence of shear advection at the solid boundaries.

To show this quantitatively, we first note that during a rapid initial phase, solute is spread uniformly along and between streamlines due to shear dispersion [2, 5]. In order to formalize this observation, we consider the streamline coordinates (ψ, ϕ), where $\psi(\mathbf{x})$ is the stream function, so that

$$\mathbf{v}(\mathbf{x}) = \left[-\frac{\partial}{\partial y}, \frac{\partial}{\partial x}\right]^{\top} \psi(\mathbf{x}), \tag{3}$$

the superscript $\top$ denotes the transpose and $\nabla\psi(\mathbf{x}) \cdot \mathbf{v}(\mathbf{x}) = 0$. Furthermore, $\nabla\phi(\mathbf{x}) \cdot \mathbf{v}(\mathbf{x}) = v(\mathbf{x}) \equiv |\mathbf{v}(\mathbf{x})|$. Thus, the coordinate ψ labels the streamline and ϕ denotes the distance along the streamline. We write $c(\mathbf{x}, t) = c[\psi(\mathbf{x}), \phi(\mathbf{x}, t)]$ and use for simplicity of notation the same letter for concentration in Cartesian and streamline coordinates. The concentration is constant along streamlines, this means that $c(\mathbf{x}, t)$ can be represented by its average along a closed streamline as

$$c(\mathbf{x}, t) = \overline{c}(\psi, t) = \frac{1}{T(\psi)} \oint \frac{d\phi}{v(\psi, \phi)} c(\psi, \phi, t), \qquad T(\psi) = \oint \frac{d\phi}{v(\psi, \phi)}. \tag{4}$$

The integral is along the closed contour $\psi = \psi(\mathbf{x})$. Note that division by v in the integrand accounts for the variable spacing between streamlines because the amount of solute between streamlines is constant. We now average the advection-diffusion equation (2) along a streamline and use (4), which gives

$$T(\psi) \frac{\partial \overline{c}(\psi, t)}{\partial t} - D \frac{\partial}{\partial \psi} \int d\mathbf{x} \nabla^2 \overline{c}[\psi(\mathbf{x}), t] H[\psi - \psi(\mathbf{x})] = 0, \tag{5}$$

where we used the identity

$$\frac{\partial}{\partial \psi} \int d\mathbf{x} F(\mathbf{x}) H[\psi - \psi(\mathbf{x})] = \int d\mathbf{x} F(\mathbf{x}) \delta[\psi - \psi(\mathbf{x})] = \oint \frac{d\phi}{v(\psi, \phi)} F(\psi, \phi). \tag{6}$$

where we used the fact that $v = |\nabla\psi|$. Using the Gauss theorem, the integral term on the right side of Eq. (5) can be written as

$$\int d\mathbf{x} \nabla^2 \overline{c}[\psi(\mathbf{x}), t] H[\psi - \psi(\mathbf{x})] = \oint d\phi \mathbf{n} \cdot \nabla \overline{c}[\psi(\mathbf{x}), t] = \oint d\phi \mathbf{n} \cdot \nabla\psi(\mathbf{x}) \frac{\partial}{\partial \psi} \overline{c}(\psi, t). \tag{7}$$

Thus, Eq. (5) becomes

$$T(\psi) \frac{\partial \overline{c}(\psi, t)}{\partial t} - D \frac{\partial}{\partial \psi} \left[C(\psi) \frac{\partial}{\partial \psi} \overline{c}(\psi, t) \right] = 0, \tag{8}$$

where we defined

$$C(\psi) = \oint d\phi v(\psi, \phi). \tag{9}$$

In order to continue we need to specify $T(\psi)$, which is the advection time along a closed contour ψ and $C(\psi)$, the circulation of the velocity field. If both quantities where constant, Eq. (5) would describe a simple diffusion equation and the trapping time distribution would scale as $\phi(t) \sim t^{-3/2}$. Here this is different. We approximate the two quantities by noting that the main contributions to $T(\psi)$ come from the vertical sections close to the no-slip boundary. There, we approximate the streamfunction by $\psi(x) = \sigma x^2/2$, where σ is the shear rate. This implies that $v(\psi) \sim \sqrt{\psi}$ and $T(\psi) \sim \psi^{-1/2}$. The circulation $C(\psi)$ is dominated by sections along the horizontal boundaries, close to which the velocity field is approximately constant. This means $C(\psi) \approx C_0$. Equation (8) can then be written as

$$T(\psi)\frac{\partial\bar{c}(\psi,t)}{\partial t} - D_0\frac{\partial^2}{\partial\psi^2}\bar{c}(\psi,t) = 0, \tag{10}$$

where $D_0 = DC_0$. We note that (10) is equivalent to the Langevin equation

$$d\psi = \sqrt{D_0\frac{dt}{T(\psi)}}\xi(t), \tag{11}$$

where $\xi(t)$ is a Gaussian white noise. We define the operational time $ds = dt/T(\psi)$ and write

$$d\psi = \sqrt{D_0 ds}\xi(s), \qquad dt = T(\psi)ds. \tag{12}$$

Thus, the time $t(s)$ is given by

$$t(s) = \int_0^s ds' T[\psi(s')] \approx \int_0^s ds' \langle T[\psi(s')]\rangle, \tag{13}$$

where the angular brackets denote the noise average. As $\psi(s)$ describes a Brownian motion, we obtain the scaling

$$\langle T[\psi(s)]\rangle \sim \int_0^\infty d\psi\psi^{-1/2}\frac{\exp[-\psi^2/(4D_0 s)]}{\sqrt{4\pi D_0 s}} \sim s^{-1/4}, \tag{14}$$

which implies that

$$t(s) \sim s^{3/4}, \tag{15}$$

i.e., time increases slower than linearly with operational time.

A. Trapping times

We want to determine the distribution of trapping times, which implies the distribution of $t(s)$ such that s is the operational time to escape the roll, this means to return to the streamline $\psi = 0$ after entering the role at $s = 0$. This means we need to determine the return time PDF. As this problem is not well-defined in continous space, we consider instead the first passage time distribution from a position ψ to the edge. For $\psi \ll 1$, this approximates the return time distribution. Since $\psi(s)$ describes a Brownian motion, the first passage time distribution from a position ψ to the edge of the role can, for times small comapred to the diffusion time across the roll, be approximated by the inverse Gaussian

$$g(s,\psi) = \frac{\psi\exp\left(-\frac{\psi^2}{4Ds}\right)}{\sqrt{2\pi D s^3}}. \tag{16}$$

This means, it scales as $g \sim s^{-3/2}$. The trapping time distribution $\phi(t)$ is obtained from $g(s)$ by variable transform

$$\phi(t)dt = g[s(t)]\frac{ds(t)}{dt}dt. \tag{17}$$

This implies for $s(t) \sim t^{4/3}$ and $g(s) \sim s^{-3/2}$ the scaling

$$\phi(t) \sim t^{-5/3}. \tag{18}$$

This behavior is valid as long as the solute is distributed along the outer streamlines of the vortex. For times larger than the diffusion time $\tau_D = R^2/D$ across the extension R of the roll, $\phi(t)$ is decaying exponentially fast.

B. Residence times

In order to obtain the residence time distribution, we consider a uniform distribution of particles inside the role. Note that a uniform distribution $p_0(x) = 1$ in equidistant space x implies in streamline coordinates that

$$p_0(\psi) \sim \psi^{-1/2}. \tag{19}$$

Thus, we obtain the residence time distribution by integration of (16) because it describes the residence time distribution of particles that originate from ψ. Thus, we obtain

$$G(s) = \int d\psi \psi^{-1/2} \frac{\psi \exp\left(-\frac{\psi^2}{4Ds}\right)}{\sqrt{2\pi D s^3}} = \int d\psi \psi^{1/2} \frac{\exp\left(-\frac{\psi^2}{4Ds}\right)}{\sqrt{2\pi D s^3}} \sim s^{-3/4}. \tag{20}$$

Thus, the residence time distribution behaves as

$$\Phi(t)dt = G[s(t)] \frac{ds(t)}{dt} dt. \tag{21}$$

The scaling of Φ is obtained substituting eq. (15) and eq. (20) into eq. (21), leading to

$$\Phi(t) \sim t^{-2/3}. \tag{22}$$

This is the scaling observed for the residence time from the experimental and numerical data.

IV. SUPPLEMENTARY INFORMATION IV

The breakthrough curves are fully determined by the particle retentention at the first step. Thus, we model the breakthrough curves through the CTRW

$$x_{n+1} = x_n + \ell_c + \sqrt{2D^* \tau_n} \xi_n, \qquad t_{n+1} = t_n + \tau_n. \tag{23}$$

With the distribution $p_0(x,t)$ of initial particle positions and times (x_0, t_0) given by

$$p_0(x,t) = \frac{1}{L} \mathbb{I}(0 < x < L) \left[(1-\alpha)\delta(t) + \alpha \Phi(t)\right]. \tag{24}$$

where α is the proportion of particles initially located in the DEPs, and $\mathbb{I}(\cdot)$ is an impulse function, which is 1 if its argument is true and 0 else. This means that the initial positions x_0 are uniformly distributed in space, and the residence time within a DEP at the initial position x_0 is given by a random time t_0. Its distribution is given by

$\Phi(t)$, which is the residence time within a DEP of depth $\Lambda\lambda_m$ and so it is the Γ distribution observed with our direct numerical simulation

$$\Phi(t) = \frac{(t/\tau_D)^{-2/3}\exp(-t/\tau_D)}{\Gamma(1/3)}. \tag{25}$$

whose -2/3 scaling was we determined in the previous section. The time increment τ_n is set constant and equal to the advection time over the distance ℓ_c as $\tau_n = \Delta t = \ell_c/u_m$ with u_m the average velocity in percolating pores. The random displacement ξ_n is a Gaussian random variable with 0 mean and unit variance, and D^* is the fitted dispersion coefficient of the transmitting pore network.

In order to determine the breakthrough curve at all times for this CTRW model, we first consider the BTC for particles that originate at a distance x from the outlet. If the particle is not in a DEP, the time to reach the outlet is distributed according to the inverse Gaussian distribution [6]

$$f_0(t,x) = \frac{x\exp\left[-\frac{(x-v_m t)^2}{4D^* t}\right]}{\sqrt{2\pi D^* t^3}} \tag{26}$$

because transport in this CTRW model is given by advection and dispersion. If the particle is initially trapped, the time to arrive at the outlet is $t_0 + t_f$, where t_f is distributed according to the inverse Gaussian (26) because transport is due to advection and dispersion once the particle has been released from the DEP. Thus, the BTC for particle originating at a distance x from the outlet can be written as

$$f(t,x) = (1-\alpha)f_0(t,x) + \alpha\int_0^t dt' f_0(t-t',x)\Phi(t'). \tag{27}$$

The full breakthrough curve $F(t)$ is obtained by integration of $f(t,x)$ over the full range L such that

$$F(t) = \frac{1}{L}\int_0^L dx f(t,x). \tag{28}$$

In order to extract the tailing behavior, we note that $f(t,x)$ is peaked about $t = x/v$ so that we can write

$$f(t,x) = (1-\alpha)f_0(t,x) + \alpha\Phi(t - x/v) \tag{29}$$

Thus, we can write for the breakthrough curve

$$F(t) = (1-\alpha)\frac{1}{L}\int_0^L dx f_0(t,x) + \alpha\frac{1}{L}\int_0^L dx\Phi(t - x/v) \tag{30}$$

For times $t \gg L/v$, we obtain

$$F(t) = (1-\alpha)\frac{1}{L}\int_0^L dx f_0(t,x) + \alpha\Phi(t) \tag{31}$$

V. SUPPLEMENTARY INFORMATION V

The concentration of colloids after the saturation phase, that lasted 24 hours, resulted non-homogeneously distributed across the medium. To quantify this distribution in terms of α, we consider a representative section (15 mm $\times$ 4 mm) from the experiment and measure the number of suspended (mobile) colloids using the method described in Methods *e*. Then, using the corresponding segregation map (see Method *a*), we compute the fraction of colloids located in DEP areas (see Figure 9): the TPs have a slightly smaller colloids concentration with respect to the DEPs, resulting in 22% of the total suspended colloids accumulated in the DEPs and 78% of the colloids in the TPs (while DEPs represent the 9% of the total volume of the porous system and the TPs represent the 91% of it). This means that the fraction of colloids initially located within a DEP is $\alpha = 0.22$ and, as a consequence, $1 - \alpha$ is the fraction of colloids initially located within a TP. We took this α value into consideration to implement the CTRW model for colloids transport and the BTC as discussed in section IV.

References

[1] F. J. H. Hol and C. Dekker. Zooming in to see the bigger picture: Microfluidic and nanofabrication tools to study bacteria. Science, 346(6208):1251821, 2014.

[2] W. Young, A. Pumir, and Y. Pomeau. Anomalous diffusion of tracer in convection rolls. Physics of Fluids A: Fluid Dynamics, 1(3):462–469, mar 1989.

[3] Jean-Philippe Bouchaud and Antoine Georges. Anomalous diffusion in disordered media: Statistical mechanisms, models and physical applications. Physics Reports, 195(4-5):127–293, November 1990.

[4] Sidney Redner. A Guide to First-Passage Processes. Cambridge University Press, August 2001.

[5] P. B. Rhines and W. R. Young. How rapidly is a passive scalar mixed within closed streamlines? Journal of Fluid Mechanics, 133:133–145, August 1983.

[6] Marco Dentz, Tanguy Le Borgne, Andreas Englert, and Branko Bijeljic. Mixing, spreading and reaction in heterogeneous media: A brief review. Journal of Contaminant Hydrology, 120-121:1–17, 2011.

[7] Pietro de Anna, Amir A. Pahlavan, Yutaka Yawata, Roman Stocker, and Juanes Ruben. Chemotaxis under flow disorder shapes microbial dispersion in porous media. Nat. Phys., 17:68–73, 2021.

[8] T. Birdal. Maximum Inscribed Circle using Distance Transform.

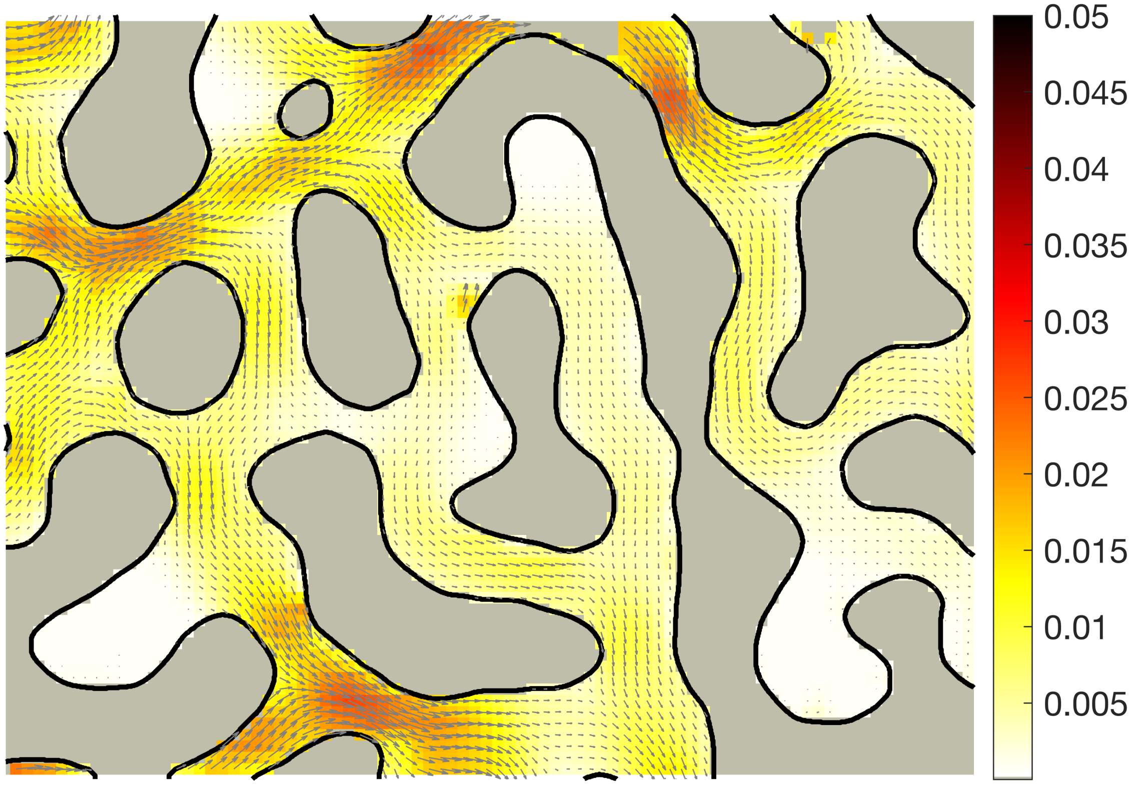

FIG. 1. **Velocity field measured via particle image velocimetry (PIV):** Absolute value of the velocity field (mm/s) superposed with velocity vectors of a portion of our system. The PIV experiment is performed following [7], seeding with 1 μm beads in water flowing at overall flow rate $Q = 0.5\mu L/min$.

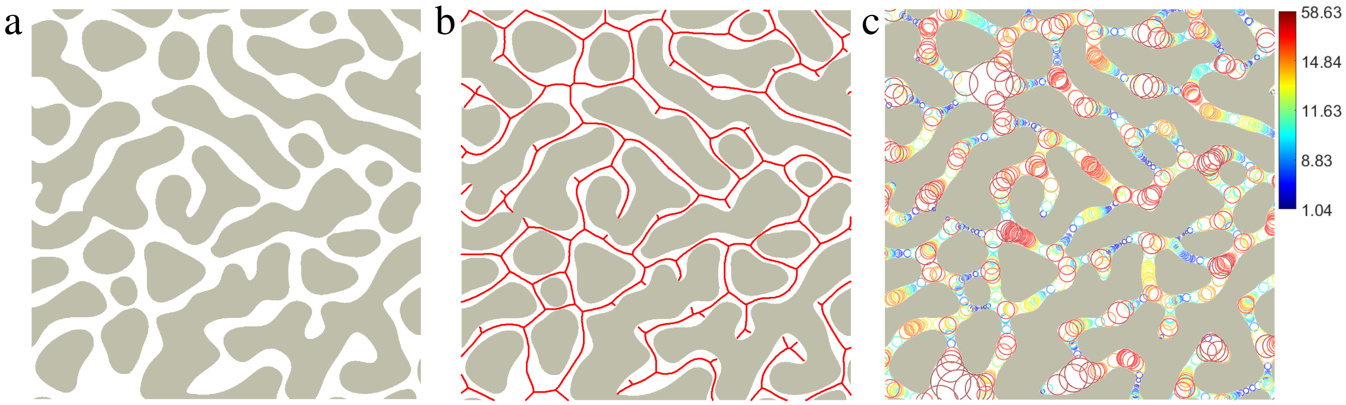

FIG. 2. **Method of maximum inscribed circle (MIC) to compute the pore-size distribution across the porous medium: a,** binary image of a small sub-section of the model porous medium with white and gray colors representing the pore-space and the constitutive grains, respectively, **b,** pixel location of the skeletonized structure (red lines) of the pore-space region, and **c,** circles fitted within the pore-space region using maximum inscribed circle method [8] and color-coded with their radii (μm).

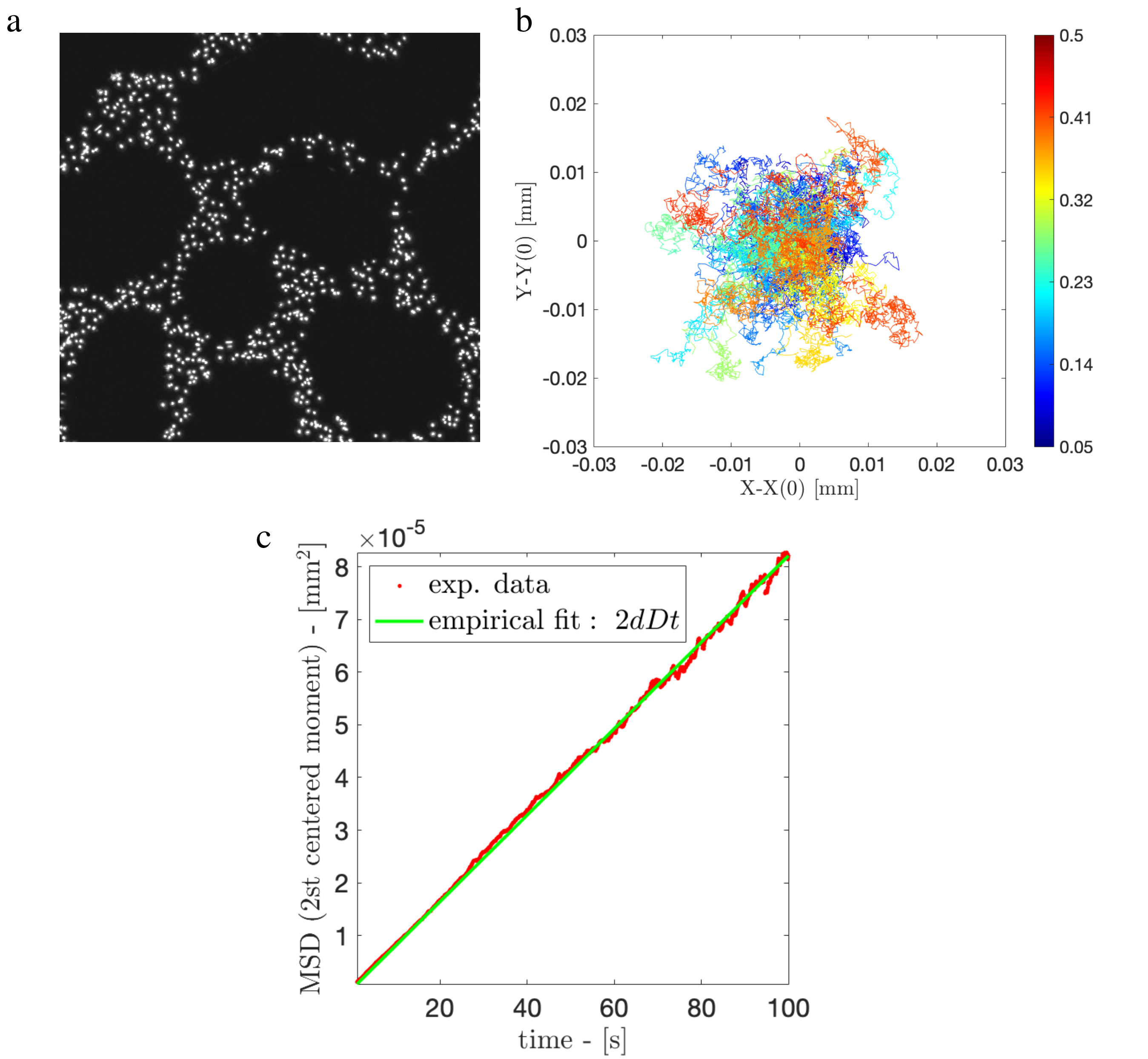

FIG. 3. **Diffusivity of suspended colloids in confined hyperuniform porous medium: a,** Fluorescence microscopy image of 0.5 μm DAPI particles; **b** sample trajectories subtracted by their initial coordinates at $t = 0$ and color-coded with the cumulative displacement (mm); **c** Temporal evolution of the mean-square displacement (MSD) averaged over 7296 trajectories. The fitted line ($2dDt$, where $d = 3$ is the dimensionality of the system) measures the diffusion coefficient as, $D = 1.4 \times 10^{-7}\mathrm{mm}^2/\mathrm{s}$.

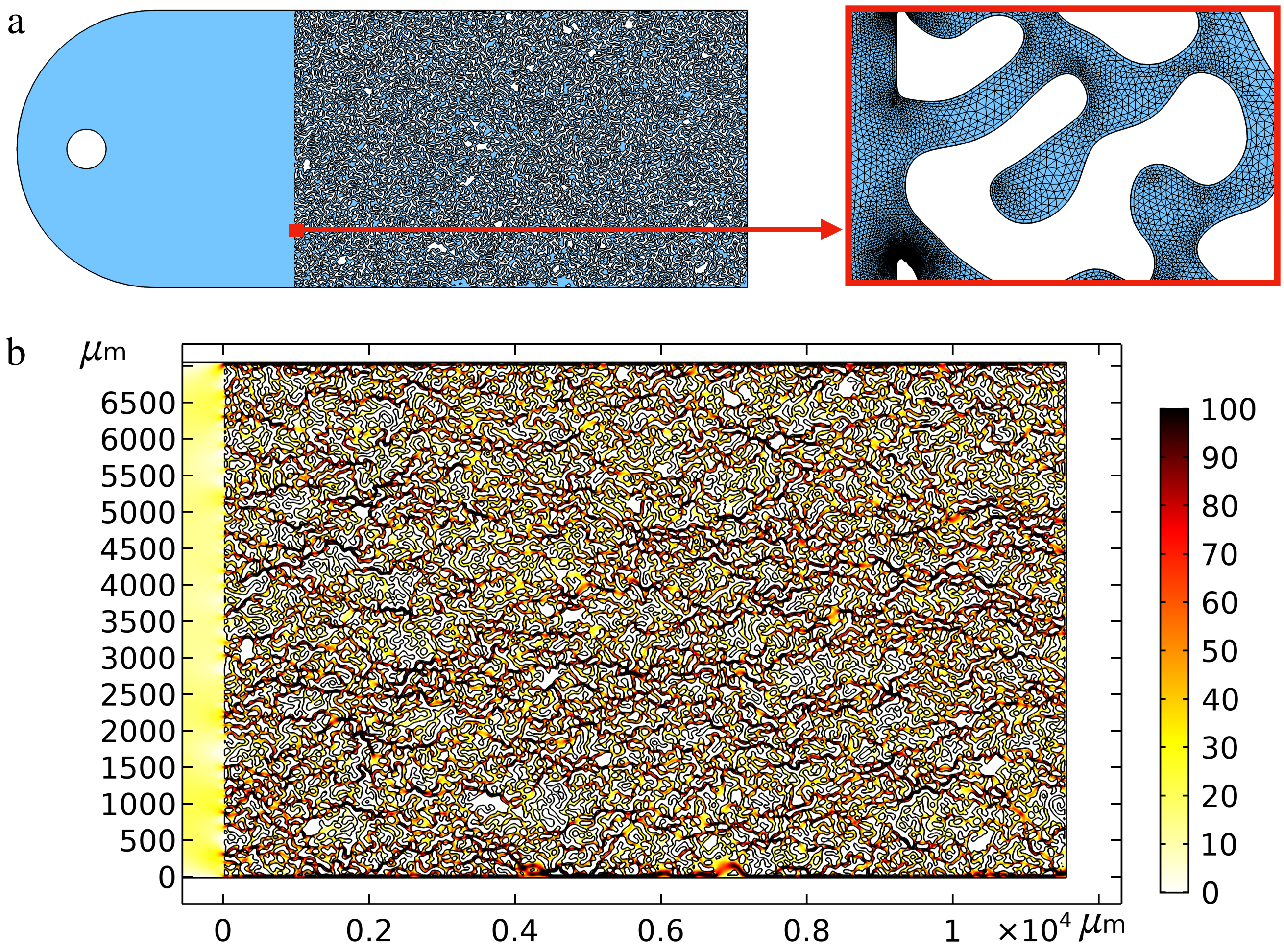

FIG. 4. **Eulerian velocity field numerical solution of the Stokes flow equations: a,** Computation domain that contains a 2D porous medium of length 11 mm and width 7 mm. Inset shows an enlarged subsection of the discretized domain having an adaptive tetrahedral mesh with minimum exlement size of 0.14 microns near each rigid surface, such that the boundary layers are well resolved. **b,** Magnitude of computed Eulerian velocity (μm/s).

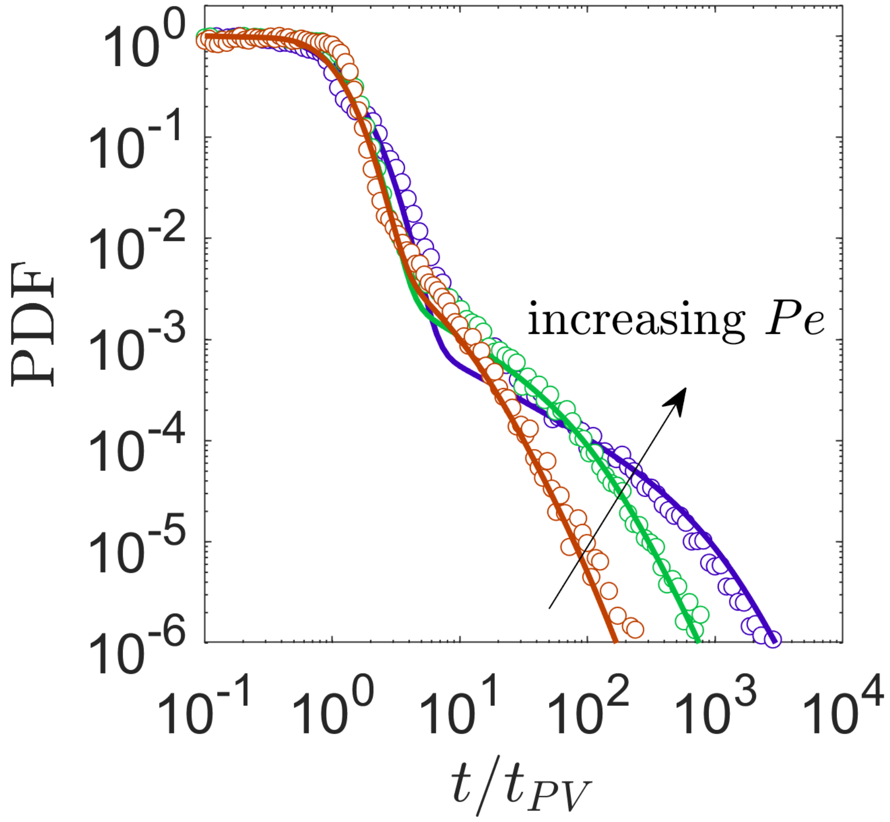

FIG. 5. **Effect of Péclet number on BTC**: Probability density function (PDF) of particle escape time (equivalent to the BTC) versus normalized time (t/t_{PV}) for Péctlet number, $Pe = 68$, 680 and 6800 obtained from tracking 10^5 homogeneously distributed particles in the simulated velocity field (symbols) and the analytical (CTRW) model (solid line). The Péclet number is varied by changing D across three orders of magnitude.

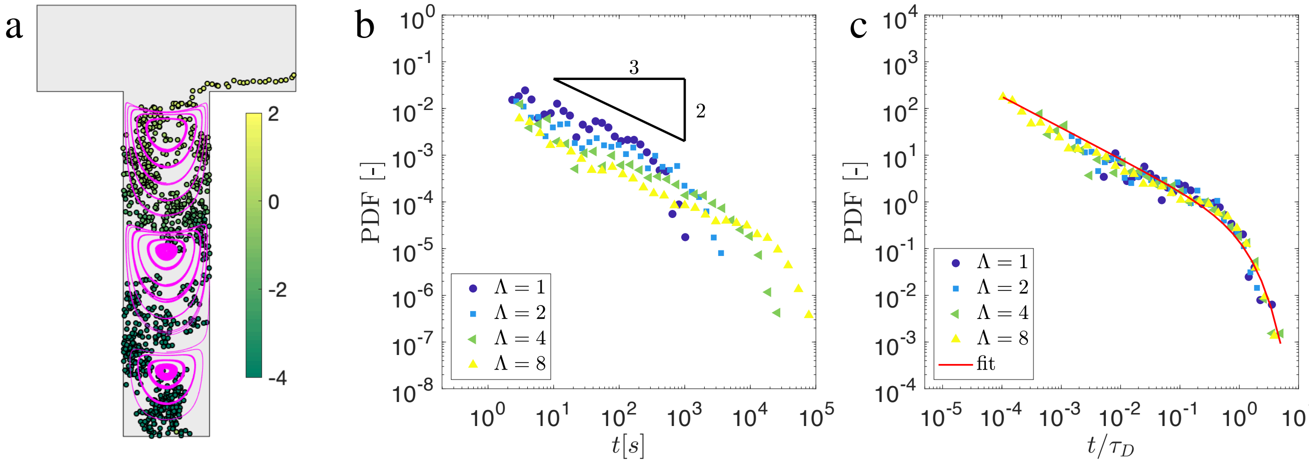

FIG. 6. **Simplified 2D model that captures the particle escape time from the dead-end pores**: **a,** Numerically simulated trajectory (color-coded with local Peclet number $Pe^* = \lambda_m v_p / D_m$) of a particle escaping from the bottom of a 2D rectangular cavity (aspect ratio, $\Lambda = 4$) connected to a channel. A series of closed streamlines highlights the vortex flow structure inside the cavity, and **b,** probability density function (PDF) of escape time of particles of a single cavity for $\Lambda = 1, 2, 4, 8$, and **c,** the same as **b** with time rescaled with diffusion time-scale $\tau_D = (\lambda_m \Lambda)^2 / D$.

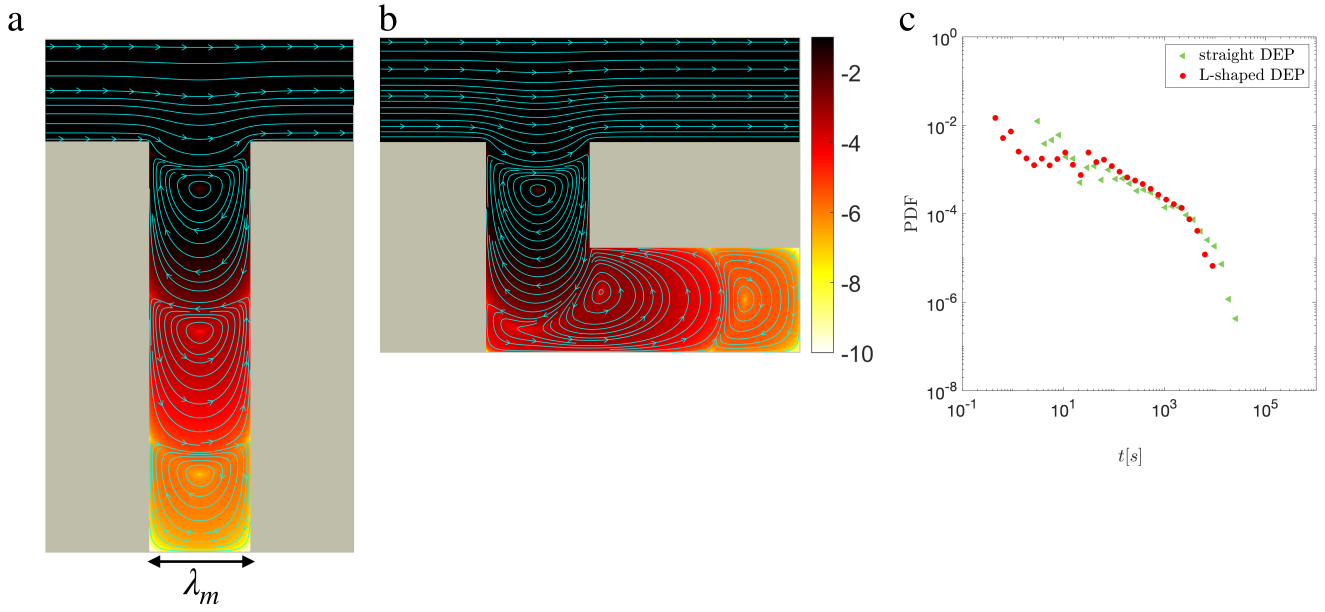

FIG. 7. **Distribution of particle escape time is independent of tortuosity of the dead-end pore.** Modulus of velocity [mm/s] in log-scale superposed with selected streamlines in a two-dimensional **a,** straight and **b,** tortuous dead-end pore with aspect ratio $\Lambda = 4$ connected to a channel, and **c,** the corresponding probability density function (PDF) of escape time of particles.

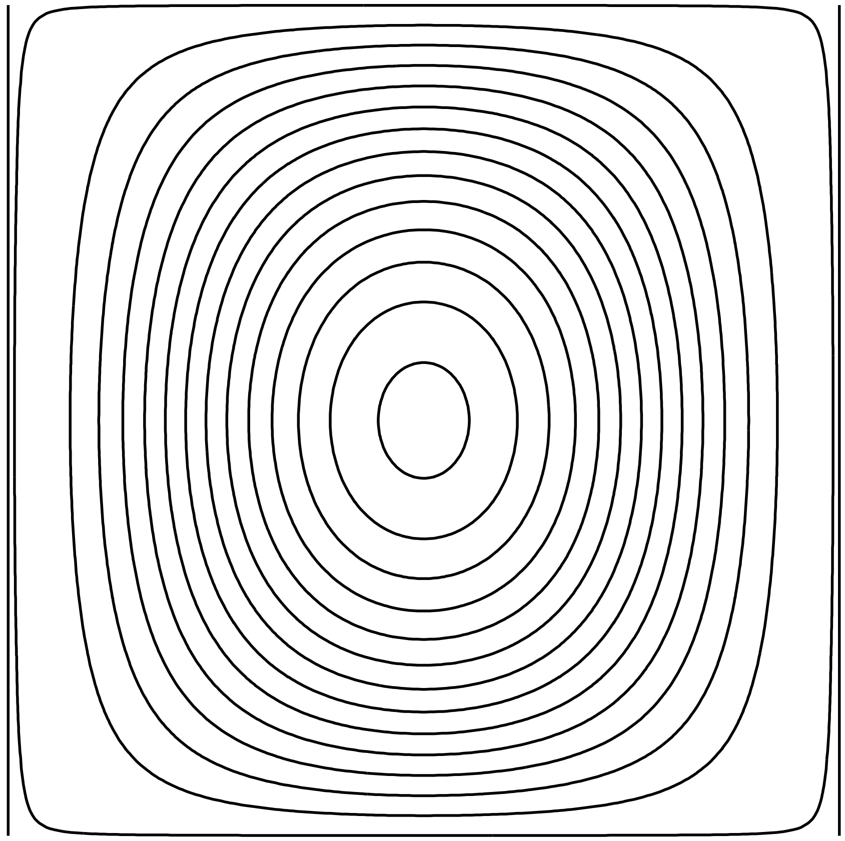

FIG. 8. Streamlines corresponding to the streamfunction $\psi(\mathbf{x}) = 16\sin(\pi y)x^2(x-1)^2$ [2], which is characterized by no-slip at the vertical boundaries at $x = 0, 1$. The spacing of the streamlines decreases with distance from the vertical boundaries, while it is approximately constant at the horizontal boundaries.

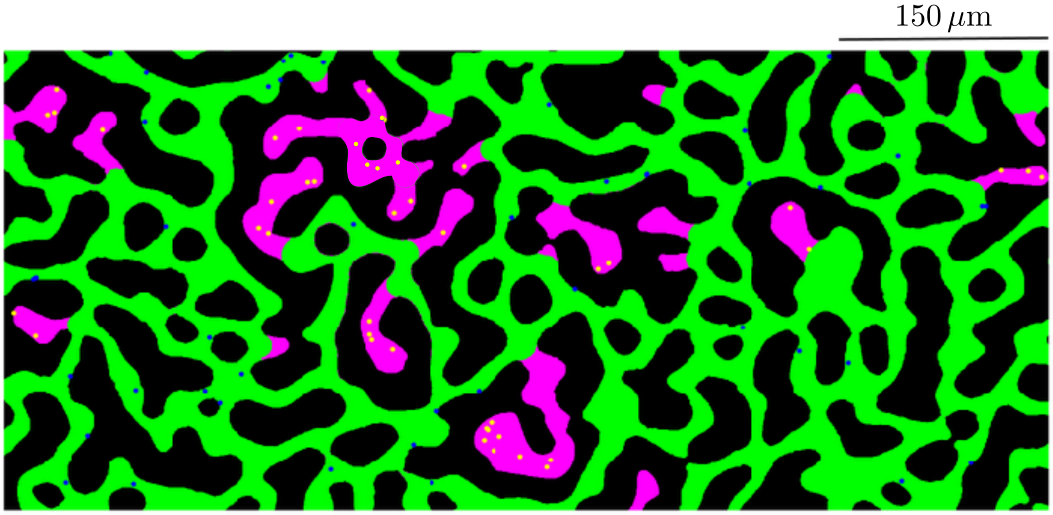

FIG. 9. **Relative distribution of suspended particles between DEP and FC in experiment:** Location of suspended particles inside the dead-end pores (yellow dots in magenta regions) and transmitting pores (blue dots in green regions) in a subsection of the porous medium. The fraction of total particle counts is $\alpha = 0.22$ in DEP and $1 - \alpha = 0.78$ in TP.
